# Bibliography

**Published:** 1891-03

**Authors:** 


### BIBLIOGRAPHY.

CATCHING'S COMPENDIUM OF PRACTICAL DENTISTRY FOR 1890. By B. H. Catching, D.D.S., Editor and Publisher, Atlanta, Ga.

This work has been looked forward to with much interest, as it seemed to promise to fill a place in dental literature at once important and necessary, both to the busy practitioner and to the student.

The labor required to collate facts is one of the serious duties of editorship, and to the conscientious writer it is of vital consequence to be sure of his authorities. This labor Dr. Catching has tried to lessen. That he has measurably succeeded in this must be conceded.

No one unfamiliar with this kind of work can realize the patience required to secure and condense facts that nothing of real value is omitted. It is an almost impossible task.

In the preface the editor says, "In this labor I have been guided by nothing but a desire to benefit dentistry." Those who know Dr. Catching personally are quite well aware that this is absolutely true, and in this effort he has had the sympathy and, as far as possible, the co-operation of the best elements in the dental profession.

The collector of such a body of facts cannot, in the nature of his subject, be responsible for the originality of the quotations. He is necessarily obliged to accept them as found. The collection will, however, be the means for comparison, and will have a value not thought of by the compiler, of enabling original workers to detect the literary piracy becoming most disagreeably prevalent in our profession.

The items selected for quotation would have greater value if they had in addition to the names the number of the journal from which they were taken. As the compendium is at present arranged it may lead to error and to credit where credit does not belong.

This defect does not interfere with its value to the practical man, who naturally has no interest in searching for the origin of things, but has in the new idea that will help him in his daily work. For such a person the condensed form adopted for this book will be a permanent and ever present friend.

In the stress of work thrown on the editor in this compilation there has, unfortunately, been some lack of attention to details, resulting in errors which will doubtless be corrected in future editions. Errors in spelling of proper names are frequent.

As a compendium is one of the books greatly needed in our profession, we hope the editor will be sustained in the effort. The value of a summary of each year's work in the profession, a condensed form always at hand for ready reference, can hardly be over estimated. The excerpts should be judiciously pruned of all unnecessary verbiage and all false assumptions. This done, no work would be of more value in the library. With the experience obtained in this first attempt and the evidence given of ability in this direction, we have no doubt but that the present editor will supply this demand in future editions.

THE DENTAL LABORATORY: A MANUAL OF GOLD- AND SILVER-PLATE WORK FOR DENTAL SUBSTITUTES, CROWNS, ETC., TO WHICH IS ADDED MANIPULATIONS IN VULCANITE AND CELLULOID, ETC. By Theodore F. Chupein, D.D.S. Published by Johnson & Lund, Philadelphia, 1890.

This work of one hundred and twenty pages, by Dr. Chupein, contains in a condensed form more valuable practical suggestions than in some similar volumes of a more pretentious character. It has been written by one closely familiar with the details of laboratory practice for many years. The name of the author became familiar to the older workers in dentistry through his valuable contributions, and it is pleasant to find that he has not lost in the intervening years the love for mechanical work, so much neglected and so little appreciated by a certain class in the profession. He has, therefore, performed a valuable service in giving his experience a permanent form.

The author commences very properly with the consideration of metal work, the knowledge of which was almost lost upon the introduction of the rubber base; but which, happily, is becoming again part of the daily experience of students in the preparation of "crown and bridge" substitutes.

To these the suggestions and clear descriptions contained in this book will be of great aid, as it has been prepared with the view to meet all the minor difficulties likely to be met with by the beginner.

The author has shown a remarkable ability to place himself in the position of the student, and enters into the details of manipulation with an enthusiasm most commendable. For the lack of this practical attention to details, many of the works of prosthetic dentistry have failed to meet the needs of the beginner, for it is in trifles that he will encounter his greatest difficulties.

The volume is clearly and properly illustrated, and it can cordially be recommended to every young practitioner as the book to keep on his laboratory table for ready reference until the subject is mastered.

UEBER DIE BEHANDLUNG CARIÖSER APPROXIMALFLÄCHEN AN MOLAREN UND BICUSPIDATEN. VON DR. THEODOR FRICK. Zurich.

This is the paper mentioned in the proceedings of the Swiss Odontological Society in the February number. It is an excellent statement of the treatment of approximal cavities, and fully in accord with modern dental thought and practice.

